# AI Applications for Chronic Condition Self-Management: Scoping Review

**DOI:** 10.2196/59632

**Published:** 2025-04-08

**Authors:** Misun Hwang, Yaguang Zheng, Youmin Cho, Yun Jiang

**Affiliations:** 1 School of Nursing University of Michigan Ann Arbor, MI United States; 2 Rory Meyers College of Nursing New York University New York, NY United States; 3 College of Nursing Chungnam National University Daejeon Republic of Korea

**Keywords:** artificial intelligence, chronic disease, self-management, generative AI, emotional self-management

## Abstract

**Background:**

Artificial intelligence (AI) has potential in promoting and supporting self-management in patients with chronic conditions. However, the development and application of current AI technologies to meet patients’ needs and improve their performance in chronic condition self-management tasks remain poorly understood. It is crucial to gather comprehensive information to guide the development and selection of effective AI solutions tailored for self-management in patients with chronic conditions.

**Objective:**

This scoping review aimed to provide a comprehensive overview of AI applications for chronic condition self-management based on 3 essential self-management tasks, medical, behavioral, and emotional self-management, and to identify the current developmental stages and knowledge gaps of AI applications for chronic condition self-management.

**Methods:**

A literature review was conducted for studies published in English between January 2011 and October 2024. In total, 4 databases, including PubMed, Web of Science, CINAHL, and PsycINFO, were searched using combined terms related to self-management and AI. The inclusion criteria included studies focused on the adult population with any type of chronic condition and AI technologies supporting self-management. This review was conducted following the PRISMA-ScR (Preferred Reporting Items for Systematic Reviews and Meta-Analyses Extension for Scoping Reviews) guidelines.

**Results:**

Of the 1873 articles retrieved from the search, 66 (3.5%) were eligible and included in this review. The most studied chronic condition was diabetes (20/66, 30%). Regarding self-management tasks, most studies aimed to support medical (45/66, 68%) or behavioral self-management (27/66, 41%), and fewer studies focused on emotional self-management (14/66, 21%). Conversational AI (21/66, 32%) and multiple machine learning algorithms (16/66, 24%) were the most used AI technologies. However, most AI technologies remained in the algorithm development (25/66, 38%) or early feasibility testing stages (25/66, 38%).

**Conclusions:**

A variety of AI technologies have been developed and applied in chronic condition self-management, primarily for medication, symptoms, and lifestyle self-management. Fewer AI technologies were developed for emotional self-management tasks, and most AIs remained in the early developmental stages. More research is needed to generate evidence for integrating AI into chronic condition self-management to obtain optimal health outcomes.

## Introduction

### Background

Chronic conditions, such as cardiovascular disease, diabetes, cancer, and chronic respiratory disease, are leading causes of death and disabilities [1]. With an aging population worldwide and increased comorbidities and complexity of care, the global burden of chronic condition management is rapidly growing [2,3]. In the United States alone, chronic conditions affected over 50% of adults in 2016, accounting for 86% of health care spending and at least 7 of the 10 leading causes of death [4]. Chronic conditions are often long term and uncertain, and patients need to take extensive responsibility for better managing their conditions [5]. It is widely accepted that self-management is essential to improve health outcomes for individuals with chronic conditions [6]. For policy makers and health care providers, self-management initiatives are increasingly recognized as an effective way to enhance health and well-being while simultaneously reducing the burdens on health care resources [7].

Patients living with chronic conditions commonly alternate exacerbations and remissions, and medical, behavioral, and emotional management are essential tasks integrated into disease self-management [8]. Medical self-management refers to adhering to prescribed medications and taking appropriate actions to manage symptoms, whereas behavioral management can involve modifying lifestyle behaviors (eg, healthy diets and physical activity). Emotional management is to cope with emotions and feelings regarding long-term chronic conditions [8]. Successful self-management, including those tasks, requires sufficient knowledge and necessary skills to manage the diseases and relevant consequences, which can be particularly challenging for most individuals [9,10].

Artificial intelligence (AI) and machine learning (ML) techniques hold the potential to overcome self-management challenges for individuals with chronic conditions. AI is defined as the technology with the ability of machines to understand, think, learn, infer, and make decisions in a similar way to human beings. ML is a subfield of AI focusing on developing algorithms and models capable of learning from data [11-13]. AI is helpful in improving the quality and access to care, reducing cost, and optimizing daily self-management when integrating with clinical information systems and patient-facing technologies [2]. AI technologies have also been reported to support chronic condition management by enabling early disease detection, improving diagnostic accuracy, and providing patient-centered care [14,15]. Multiple studies have assessed the efficacy of AI in contributing to positive health outcomes, including weight loss, controlling blood glucose, pain management, psychosocial well-being, and the quality of life by enhancing self-management of chronic conditions [16-19].

However, while AI technologies are progressing toward tailoring support for specific types of chronic conditions [20], there is a lack of understanding of the current levels of AI applications to support chronic condition self-management systematically and how AI is integrated into self-management processes and specific tasks, such as medical, behavioral, and emotional self-management. Existing literature reviews focused on developing a specific type of AI technology for certain chronic condition management outcomes (eg, glucose level prediction for managing diabetes, improving diagnostic tools for liver diseases, or severity classification of respiratory disease) [20-23]. One recent study reviewed AI applications for chronic disease management but did not focus on how AI can support patients’ needs and performance in self-management [24].

### Objectives

Thus, the objectives of this study were to provide a comprehensive overview of AI applications for chronic condition self-management, with self-management components supported by AI technologies based on tasks of medical, behavioral, and emotional self-management, and to identify the current developmental stages and knowledge gaps of AI applications for self-management of chronic conditions.

## Methods

### Study Design

This study is a scoping review of the literature conducted following the PRISMA-ScR (Preferred Reporting Items for Systematic Reviews and Meta-Analyses Extension for Scoping Reviews) guidelines [25]. The completed PRISMA-ScR checklist is described in Multimedia Appendix 1.

### Search Strategy

In total, 4 databases, including PubMed, Web of Science, CINAHL, and PsycINFO, were used to search articles published between January 2011 and October 2024 to obtain a comprehensive list of studies relevant to our research topic. The search strategies were developed based on consultation with a health sciences librarian. Two groups of search terms—*self-management* and *artificial intelligence* were used in combination with their Medical Subject Headings (MeSH) terms, keywords, and synonyms. The details of the search strategy are presented in Multimedia Appendix 2.

### Eligibility Criteria

The eligibility criteria for this scoping review are described in Textbox 1. In this review, chronic conditions are defined as those lasting >1 year and requiring ongoing medical attention or limiting activities of daily life, following the definition provided by the Centers for Disease Control and Prevention [26].

Research team members worked with a health sciences librarian on the literature search and the initial title and abstract screening. All authors (MH, YC, YZ, and YJ) evaluated the selected full texts and determined the data extraction strategies. The desired level of screening agreement among raters was set at 80% and achieved 100% after group discussion.

**Eligibility criteria for scoping review.**

**Inclusion criteria**

- Articles that applied any type of artificial intelligence (AI) technologies in self-management for chronic conditions
- Articles that targeted adults aged ≥18 years
- Articles published in English

**Exclusion criteria**

- Articles that had no component of chronic condition self-management (eg, AI for daily activities, for only diagnosing or predicting the incidence of diseases, or for specific physical outcomes)
- Articles that had no description of the component of AI
- Non–data-driven articles (eg, viewpoints, editorial comments, or review articles)
- Articles with no access to the full text

### Data Extraction and Information Synthesis

Study characteristics and information regarding AI applications in self-management were extracted from each reviewed article. Basic study characteristics included authors, year of publication, country, and target chronic condition. The types of AI technologies and their applications to support patients’ self-management tasks were extracted and reported. The tasks included 3 categories: medical, behavioral, and emotional self-management [8]. In this review, AI for medical self-management encompasses AI technology’s specific role in predicting disease processes and providing personalized suggestions or decision-making support tailored to specific conditions. Behavioral self-management encompasses AI technology’s role in monitoring and helping with lifestyle modification or providing personalized self-management suggestions. Finally, emotional self-management encompasses AI technology’s role in providing emotional support or assisting in motivation improvement. The outcomes of each AI technology were reported to review their effectiveness and impact on self-management.

In addition, we mapped included studies according to the 9 generic study types for technology evaluation as reported by Friedman and Wyatt [27] to categorize the current developmental stage of each study. Next, we applied the evaluation framework provided by Yen and Bakken [28], which is proposed based on the system developmental life cycle (Table 1) [29].

**Table 1:** Mapping artificial intelligence developmental stages with technology evaluation study types.

Developmental stage	Criteria for study classification	Fridman and Wyatt [27] study types
Stage 1	Needs assessments	Needs assessment
Stage 2	Evaluation of system validity	Design validation Structure validation
Stage 3	Evaluation of human-computer interaction	Usability test Laboratory function study Laboratory user effect study
Stage 4	Field testing; experimental or quasi-experimental designs in one setting	Field function study Field user effect study
Stage 5	Field testing; experimental or quasi-experimental designs in multiple sites	Problem impact study

## Results

### Study Selection

**Figure 1:**
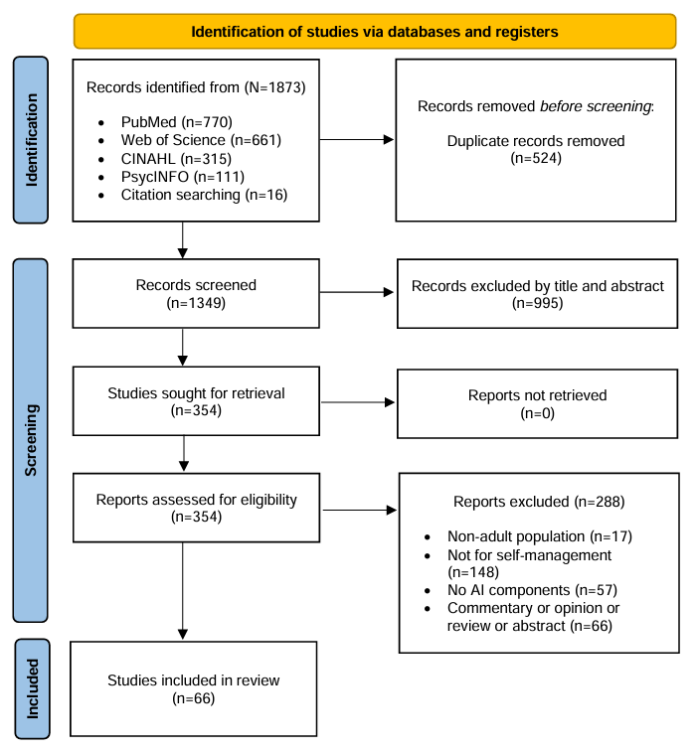
PRISMA flow diagram regarding the study selection process. AI: artificial intelligence.

Of the 1873 articles retrieved in the initial search, 524 (27.9%) duplicates were removed. After assessing the titles and abstract, 73.7% (995/1349) of articles were excluded, and a full-text review was conducted on the remaining 26.2% (354/1349) of articles. Subsequently, 81.3% (288/354) of articles were excluded based on the inclusion and exclusion criteria. The reasons for exclusion were that the reported articles were not conducted for the adult population (n=17, 4.8%); did not include self-management components (n=148, 41.8%); did not include AI components (n=57, 16.1%); or were commentary, opinion, review, or abstract (n=66, 18.6%). Consequently, 66 (18.6%) articles or studies were included in the final analysis (Figure 1).

### Study Characteristics

#### Types of Chronic Conditions

Table 2 classifies the general characteristics of each study. About a third of the studies (20/66, 30%) were conducted among patients with diabetes, including type 1, type 2, and gestational diabetes; 14% (n=9) were conducted among patients with respiratory diseases, such as chronic obstructive pulmonary disease (COPD) and asthma; 12% (n=8) were conducted among patients with cancer and chronic pain, respectively; 8% (n=5) were conducted among patients with cardiovascular diseases, including heart failure and hypertension; and 24% (n=16) were conducted among patients with other conditions, such as stroke, frozen shoulder, spinal cord injury, inflammatory bowel diseases, irritable bowel syndrome, multiple chronic conditions, ostomy, chronic kidney disease, chronic liver disease, or patients taking medications without mentioning specified chronic conditions.

**Table 2:** The classification of study characteristics (N=66).

Characteristics				Studies, n (%)		Included studies
**Publication year**						
	2011-2019			28 (42)		[30-57]
	2020-2024			38 (58)		[17-19,58-92]
**Continent**						
	North America			25 (38)		[19,30,31,39,42,43,48,51,54,57,64,69,70,72,73,76-78,81,82,84,86,88,91,92]
	South America			2 (3)		[59,89]
	Europe			25 (38)		[18,32-38,44,46,49,50,52,53,55,56,58,60,62,63,65,67,68,75,85]
	Australia			3 (5)		[17,41,83]
	Asia			11 (17)		[40,45,47,61,66,71,74,79,80,87,90]
**Type of chronic condition**						
	Diabetes			20 (30)		[17,30,40,41,44,48-50,55,60,61,67,68,71,72,74,75,82,91,92]
	Respiratory diseases			9 (14)		[31-34,46,52,58,62,69]
	Cardiovascular diseases			5 (8)		[18,35,59,76,77]
	Cancer			8 (12)		[53,79,81,84-88]
	Chronic pain			8 (12)		[19,36,43,47,63,65,73,78]
	Other conditions^a^			16 (24)		[37-39,42,45,51,54,56,57,64,66,70,80,83,89,90]
**Type of AI^b^ technologies**						
	Conversational AI (including NLP^c^)			21 (32)		[17,52-54,74-90]
	ML^d^ (multiple algorithms)			16 (24)		[18,30-39,58-62]
	Neural Network			13 (20)		[45-51,68-73]
	ML (single algorithm)			7 (11)		[40-42,63-66]
	RL^e^ (including deep RL)			4 (6)		[19,43,44,67]
	Nonspecified			5 (8)		[55-57,91,92]
**Technology developmental stage^f^**						
	System validity testing (stage 2)			25 (38)		[30-35,37,39-41,44,46,48,50,57,58,60-62,67-69,72,75,80]
	Usability testing (stage 3)			12 (18)		[45,52,54,56,76,79,81-83,87-89]
	Laboratory study (stage 3)			13 (20)		[36,38,42,43,49,53,59,64,66,70,74,78,91]
	**Field testing (stages 4 and 5)**					
		Randomized controlled trial	10 (15)		[17-19,51,63,65,71,77,84,85]	
		Quasi-experimental study	5 (8)		[47,55,73,86,90]	
		Observational study	1 (2)		[92]	

^a^Other conditions include stroke, frozen shoulder, spinal cord injury, inflammatory bowel diseases, irritable bowel syndrome, multiple chronic conditions, ostomy, chronic kidney disease, chronic liver disease, or patients taking medications without mentioning specified chronic conditions.

^b^AI: artificial intelligence.

^c^NLP: natural language processing.

^d^ML: machine learning.

^e^RL: reinforcement learning.

^f^According to the criteria given in Table 1.

#### Types of AI Technologies

Most studies (40/66, 61%) have applied ML-based algorithms to support self-management, including neural networks and reinforcement learning (RL). It was common for the studies (16/66, 24%) to compare the performances of multiple ML algorithms, such as support vector machines (SVMs), random forest (RF), naïve Bayesian, decision tree (DT), adaptive boosting, and *k*-nearest neighbors, or combine ML and deep learning (DL) algorithms for the application [18,30-39,58-62]. Fewer studies (7/66, 11%) only used 1 type of ML algorithm, such as SVM, logistic regression, DT, or case-based reasoning [40-42,63-66]. RL and deep RL were used in 4 (66%) studies [19,43,44,67]. Some (13/66, 20%) studies used neural network models for prediction [45-51,68-73]. The application of conversational AI, such as chatbots or virtual assistants, was reported in 21 (32%) studies [17,52-54,74-90]. Natural language processing (NLP), a key component of conversational AI, was solely used in 4 (6%) other studies [54,80-82]. The type of AI technologies in the other 5 (8%) studies was not specified [55-57,91,92].

#### AI Technology Development Stage

More than one-third of studies (25/66, 38%) were in stage 2, which involves system validity testing. Similarly, another third (25/66, 38%) were included in stage 3, which includes either usability testing (12/25, 48%) or laboratory function or user effect testing (13/25, 52%). The remaining studies (16/66, 24%) were categorized into stage 4 or stage 5, conducting field testing, experimental study, or quasi-experimental study in the real world. Specifically, 10 (15%) studies conducted randomized controlled trials (RCTs) [17-19,51,63,65,71,77,84,85]. In total, 6 (9%) studies used quasi-RCTs [55], one-group pretest-posttest designs [47,73,86,90], or an observational study [92].

### Self-Management Tasks by AI Functions and Developmental Stages

#### Overview

Table 3 describes self-management tasks (medical, behavioral, and emotional self-management) by categorized functions of AI technologies and the technology developmental stage of each study. Table 4 provides a detailed summary of the studies included.

**Table 3:** Self-management tasks and developmental stage.

Self-management tasks and category of functions		Developmental stage		
		Stage 2	Stage 3	Stages 4 and 5
**Medical self-management (n=45)**				
	Personalized recommendation for medication or treatment-related decision-making (n=13)	Insulin dose [44,67] The next dosage of anticoagulation [37]	Insulin dose [49] Crisis support during acute exacerbations of COPDa [52] Referral advice to manage pain [36] Medication and nutrition-specific information for chronic liver disease [83] Clinical reminders for patients with chronic kidney disease [66] Ostomy management [89]	General treatment decisions for patients with diabetes [55,92] Adjusting the modality of therapist interaction to manage pain [19] Peritoneal dialysis management [90]
	Promoting medication adherence and safety (n=8)	Monitoring medication adherence [57,75] Detecting inhaler administration [46]	Monitoring medication adherence [42] Detecting insulin administration [68] Improving medication adherence and safety [56,70]	Improving medication adherence and safety [51]
	Prediction of physiological indicators or clinical outcomes (n=19)	Predicting blood glucose levels or hypoglycemia events [30,40,48,50,60,61,72] Predicting risk of asthma or COPD exacerbation [31-34,58,62] Predicting adverse events or classification of the extent of heart failure [35]	Predicting blood glucose levels [49] Predicting risk of adverse events of heart failure [76] Identifying heart arrhythmias [59]	Predicting blood pressure [18] Predicting pain level [73]
	Cancer management (n=6)	—^b^	Cancer-related symptoms [79] Postoperative management [87] Oral anticancer agents [53,88]	Cancer-related symptoms [84] Chemotherapy-related side effects [85]
**Behavioral self-management (n=27)**				
	Provision of personalized recommendations and feedback on lifestyle and healthy behavior (n=21)	Diet, physical activity, and other lifestyles for patients with diabetes [41,75] Diet or physical activity for patients with heart failure [76] Various health behaviors for patients with multiple chronic conditions [80]	Diet, physical activity, and other lifestyles for patients with diabetes [82,91] Physical activity for patients with chronic pain [43,47] Diet for patients with chronic conditions (multiple chronic conditions, IBDc or IBSd, or chronic kidney disease) [54,64,90] Various health behaviors for patients with chronic kidney disease [66]	Diet, physical activity, and other lifestyles for patients with diabetes [17,74,92] Diet or physical activity for patients with cardiovascular diseases [18,77] Nutrition for patients with cancer [84,86] Various health behaviors for patients with chronic pain [63,65]
	Predicting and monitoring health behavior outcomes (n=8)	Symptom self-management ability [69] Treatment adherence and adherence risk [35] Prediction of ambulation status and independence [39]	Monitoring rehabilitation [38,45]	Monitoring physical activity [18,84] Monitoring diet [71]
**Emotional self-management (n=14)**				
	Providing personalized emotional support (n=9)	Encouraging expressing emotions through facial and body animations [75]	Emotional support during periods of low moods [52] Responding to patients’ distress [78] Identifying psychosocial concerns and providing recommendations [81] Building emotional attachments [53,83]	Emotional support by recognizing feelings from physiological data (voice, heart rate) [18] Enhancing psychological flexibility [73,84]
	Motivating to perform self-management activities (n=6)	Motivating to support performing self-management [17,74] Encouraging patients to perform self-management [57]	Motivating to support performing self-management [52]	Motivating and reinforcing the desired self-management activities [63,65]

^a^COPD: chronic obstructive pulmonary disease.

^b^Not available.

^c^IBD: inflammatory bowel disease.

^d^IBS: irritable bowel syndrome.

**Table 4:** Summary of included studies (N=66).

Author (year)	Country	Chronic conditions	Evaluation stage of each study	Type of AI^a^	Self-management components supported by AI.	Main results
Huang et al [41], (2015)	Australia	Diabetes	System validity testing	ML^b^ (SVM^c^)	The SVM classifier implemented in a smartphone app was trained using a fruit database consisting of 10 types of fruits. Each fruit type included 60 images. SVM classifies food types and volumes and calculates the amount of carbohydrates to help patients control their diet.	Overall accuracy of SVM classification of fruits was 90%. The average error rate was 6.86%.
Shi et al [40], (2015)	China	Diabetes	System validity testing	ML (linear regression)	The linear regression method was selected to gain a prediction model, and 2 algorithms were tested: gradient descent and normal equation. The key technique is that the system uses ML to extract the prediction model from the training sample based on user input. ML algorithm predicts postprandial glucose level by analyzing the diet of patients.	Prediction accuracy using gradient descent and the normal equation was 63% and 73%, respectively.
Sudharsan et al [30], (2015)	United States	Diabetes	System validity testing	ML (RF^d^, SVM, k-nearest neighbor, and naïve Bayes)	If a set of blood glucose values is available for a given week, it can be predicted whether the patient will have a hypoglycemic episode in the following week. ML algorithms predict a hypoglycemia event in the next 24 h using self-monitored blood glucose and medication information.	Prediction accuracy was over 90% in models using RF or SVM. RF and SVM models had a 91.7% sensitivity and 69.5% specificity. After incorporating medication information, the sensitivity and specificity were over 90%.
Faruqui et al [48], (2019)	United States	Diabetes	System validity testing	DL^e^ (recurrent neural networks)	The DL model predicts daily glucose levels based on patient health data, including glucose levels from the day before, diet, physical activity, and weight. Neural networks used multiple layers of computational nodes to model how mobile health data progressed from one day to another from noisy data.	Prediction accuracy was within 10% of the actual values based on the Clark Error Grid.
Balsa et al [75], (2020)	Portugal	Diabetes	System validity testing	Conversational AI	An intelligent virtual assistant–based system (VASelfCare) supports medication adherence and lifestyle change, including healthy diets and physical activity. Patients interact with the virtual assistant that can speak and express emotions through facial and body animations.	Participants (n=20) reported 73.75 of the system usability score on average, which is a borderline rating of excellent.
Gong et al [17], (2020)	Australia	Diabetes	Field testing (2-group RCT^f^)	Conversational AI	A conversation AI (My Diabetes Coach) provides personalized support, including blood glucose monitoring, diet, physical activity, medication, and foot care. Algorithms were tailored according to the clinical targets and recommendations provided by each participant’s health care providers.	Participants in the intervention group (n=93) and control group (n=94) reduced HbA1cg (intervention: 0.33% and control: 0.20%) compared to baseline. Health-related quality of life utility score was improved in the intervention group (P=.04).
Krishnakumar et al [74], (2021)	India	Diabetes	Laboratory function testing	Conversational AI	A conversational AI (chatbot) communicates with patients regarding diet, physical activity, and blood glucose and provides personalized feedback based on previous data. Patients can receive motivational messaging, reducing the difficulty in performing specific tasks and providing triggers needed to act. AI-powered decision support system (Wellthy CARE) enabled across the platform.	Participants reported reduced HbA1c by 0.49% (n=102), fasting blood glucose by 11 mg/dL (n=51), postprandial blood glucose by 21 mg/dL (n=51), BMI by 0.47 kg/m2 (n=59), and weight by 1.32 kg (n=59) after 4 mo.
Mitchell et al [91], (2021)	United States	Diabetes	Laboratory function testing	Others^h^	ML based on attributable components analysis identifies patterns and relationships between meals and changes in blood glucose levels. The system (GlucoGoalie) translates ML output into actionable support by generating natural language recommendations for personalized nutritional support to improve blood glucose levels.	In the goal comprehension task, participants accurately selected between 2 nutrition labels 89% of the time. When choosing between 2 meal images, their accuracy was 49%.
Thyde et al [68], (2021)	Denmark	Diabetes	System validity testing	DL (convolutional neural networks)	The DL model detects early adherence to once-daily basal insulin based on continuous glucose monitoring and injection data. Six different detection models were compared according to whether they fused expert-dependent and automatically learned features.	The 3 models based on expert-engineered features reported mean accuracies of 78.6%, 78.2%, and 78.3%. The model based on learned features reported a mean accuracy of 79.7%. The 2 models fusing expert-engineered and learned features reported mean accuracies of 79.7% and 79.8%.
Sy et al [82], (2022)	United States	Diabetes	Usability testing	NLP^i^	Patients can receive personalized recommendations reflecting their health-related social needs for self-management detected by NLP.	The overall engagement ratio with personalization was an average of 0.31, while the engagement ratio without personalization was 0.26.
Kumbara et al [92], (2023)	United States	Diabetes	Field testing (single-arm, retrospective study)	Others	An AI-based mobile app (BlueStart) provides personalized feedback and coaching to help patients track their medication, sleep, exercise, and other health behaviors. Patients can view their glucose levels and identify patterns through a continuous glucose monitoring system (Dexcom G6) that synchronizes with the app.	Participants with baseline mean glucose >180 mg/dL (18/52) demonstrated significant improvements in glycemic control after 3 mo.
Lee et al [71], (2023)	South Korea	Diabetes	Field testing (open-label multicenter RCT)	DL (convolutional neural network)	An AI-based mobile dietary management platform (Auto-Chek Care) collects data from multiple devices linked via Bluetooth and performs integrated analysis. A DL-based food recognition system (FoodLens) incorporates diet and nutritional data from photographs taken by patients into the platform.	The decreases in HbA1c from baseline to 6-mo in intervention group 1 (–0.32, SD 0.58%) and intervention group 2 (–0.49, SD 0.57%) were significantly larger than those in the control group (–0.06, SD 0.61%) Intervention groups demonstrated greater weight loss than the control group after 6 mo.
Alexiadis et al [60], (2024)	Greece, United Kingdom	Diabetes	System validity testing	DL (artificial neural network), ML (RF, SVM, and AdaBoost^j^)	The algorithms are used to perform next-day hypoglycemia prediction in daily life based on the data input from a mobile app (forDiabetes) and portable devices.	RF showed the best accuracy (0.814) and F1-score (0.812) with sensitivity (0.815) and specificity (0.824). The accuracy of other models ranges from 0.65 to 0.80.
Gong et al [61], (2024)	China	Diabetes	System validity testing	ML (logistic regression, RF, and light gradient boosting machine)	ML algorithms predict nocturnal hypoglycemia based on continuous glucose monitoring data.	The light gradient boosting machine model had the highest predictive performance: accuracy (0.801), specificity (0.802), and F1-score (0.255).
Zecchin et al [50], (2014)	Italy	Type 1 diabetes	System validity testing	DL (jump neural network)	Jump neural network means a feed-forward neural network whose inputs are connected not only to the first hidden layer but also to the output layer. Neural network predicts continuous blood glucose based on patients’ input of the carbohydrate amount.	Predictions are accurate and close to target (root mean squared error=17.6 mg/dL).
Pérez-Gandía et al [49], (2018)	Spain	Type 1 diabetes	Laboratory function testing	DL (artificial neural network)	The DSSk, named GlucoP, was designed to help patients in real-time while performing therapeutic corrective actions, including administration of insulin bolus to correct hyperglycemia or intake of carbohydrates in case of hypoglycemia. The glucose predictor is based on an artificial neural network trained with continuous glucose monitoring profiles.	After perceiving the glucose prediction, 20% of participants decided to revise their initial decision. Participants (n=12) reported positive opinions about usability (>7 on average out of 9) and described using the DSS as a pleasant experience.
Sun et al [44], (2019)	Switzerland	Type 1 diabetes	System validity testing	ML (reinforcement learning)	The ML-based ABBAl allows daily adjustment of the insulin infusion profile to compensate for fluctuations in the patient’s glucose level. ABBA provides personalized suggestions for the daily basal rate and prandial insulin doses based on the patients’ glucose level on the previous day.	ABBA significantly decreased the percentage of time in hypoglycemia and severe hypoglycemia ranges (P<.05), while the percentages of hyperglycemia were increased.
Zhu et al [67], (2020)	United Kingdom	Type 1 diabetes	System validity testing	DL and ML (reinforcement learning)	The DRLm adviser can calculate the gain of the meal insulin bolus to help users control the insulin pump or pen. The DRL adviser provides a personalized insulin bolus adviser to optimize insulin at mealtime.	DRL insulin bolus adviser improved percentage time in target scope (70-180 mg/dL) from 74.1% to 80.9% (for adults) and 54.9%-61.6% (for adolescents) while reducing hypoglycemia.
Mosquera-Lopez et al [72], (2023)	United States	Type 1 diabetes	System validity testing	DL (evidential neural network)	The DL algorithm predicts at bedtime the probability and timing of nocturnal hypoglycemia based on glucose metrics and physical activity patterns. Predictions are used to prescribe bedtime carbohydrates in a timely manner.	An area under the receiver operating curve of the model ranged from 0.71 to 0.80 to predict nocturnal hypoglycemic events.
Rigla et al [55], (2018)	Spain	Gestational diabetes	Field testing (quasi RCT)	Others	MobiGuide is an AI-based mobile system based on computer-interpretable guidelines for providing personalized decision support to patients having a DSS at the back end and a body area network on the front end. AI using DSS enables personalized decision support or feedback based on patient-reported blood glucose, ketonuria, diet, blood pressure, and physical activity without medical supervision.	Participants (n=20) reported a higher degree of compliance and satisfaction. Systolic and diastolic blood pressure were significantly lower in the intervention group (P<.001), compared to the historical cohort group.
Finkelstein and Jeong [31], (2017)	United States	Asthma	System validity testing	ML (adaptive Bayesian network, naïve Bayesian classifier, and SVM)	ML algorithms predict before an asthma exacerbation occurs based on data, including respiratory symptoms, sleep disturbances, limitation of physical activity, medication use, and measured peak expiratory flow.	3 models using naïve Bayesian, adaptive Bayesian network, and SVM predicted asthma exacerbation occurring on day 8, with the sensitivity of 0.80, 1.00, and 0.84; specificity of 0.77, 1.00, and 0.80; and accuracy of 0.77, 1.00, and 0.80, respectively.
Kocsis et al [32], (2017)	United Kingdom	Asthma	System validity testing	ML (SVM, RF, and AdaBoost)	ML algorithms–based system (myAirCoach) conducts short-term prediction of asthma control levels for real-time personalized guidance and long-term prediction of exacerbation risk.	Prediction accuracy of 3 models ranged from 0.79 to <0.86 according to tested cases.
Anastasiou et al [33], (2018)	Greece	Asthma	System validity testing	ML (AdaBoost, SVM, RF, and naïve Bayesian)	ML algorithms–based system (myAirCoach) conducts an estimation of the risk of asthma exacerbation 7 d ahead based on data.	Prediction accuracy of 4 models was good for predicting exacerbation 7 d in advance. RF algorithm had an overall greater accuracy, and SVM was effective for predicting the true positive cases.
Easton et al [52], (2019)	United Kingdom	COPD^n^	Usability testing	Conversational AI	A conversational AI (Avachat) provides crisis support during acute exacerbation periods and information at the time of diagnosis. Patients can be motivated to perform self-management and receive emotional support during periods of low moods.	The median system usability score was 73.75 out of 100 (n=8).
Kocsis et al [34], (2019)	Greece	Asthma	System validity testing	ML (SVM, RF, AdaBoost, and Bayesian Network)	ML algorithms–based system (myAirCoach) conducts short-term prediction of asthma control levels and long-term prediction of exacerbation risks in the myAirCoach decision support.	Prediction accuracy of the RF algorithm was the best at 0.80. SVM and RF classifiers were superior in all cases compared to the AdaBoost and naïve Bayesian.
Pettas et al [46], (2019)	Greece	Asthma and COPD	System validity testing	DL (recurrent neural networks)	The DL model, using recurrent neural networks with long short-term memory units and spectrogram features, was tested to monitor medication adherence. The DL model-based audio signal segmentation approach monitors medication adherence by detecting pressurized metered-dose-inhaler audio events.	Prediction accuracy of the DL model with intrasubject and intersubject settings ranged from 0.92 to 0.94.
Tsang et al [58], (2020)	United Kingdom	Asthma	System validity testing	ML (decision tree, logistic regression, naïve Bayesian, and SVM)	ML algorithms classify whether a patient is stable or unstable and allow early warning of asthma aggravation.	Prediction accuracy of both logistic regression and naïve Bayesian algorithm was the best at 0.87.
Bugajski et al [69], (2021)	United States	COPD	System validity testing	DL (artificial neural network)	Data regarding symptoms and self-management abilities were entered into an artificial neural network using a 3-layer model (input, hidden, and output layers). To ensure stability and reproducibility, a standard feed-forward, multilayer perceptron neural network was used. The model classified participants into their respective self-management ability tertiles (low, medium, or high).	Prediction accuracy of artificial neural network to predict self-management ability was 0.94 with 21 d of consecutive data.
Glyde et al [62], (2024)	United Kingdom	COPD	System validity testing	ML (AdaBoost and EasyEnsemble classifier)	An interactive cloud-based digital app (myCOPD) predicts exacerbations before 1-8 d based on patient-reported data.	An AdaBoost model showed 35% sensitivity and 89% specificity. The EasyEnsemble classifier showed 67% sensitivity and 65% specificity.
Tripoliti et al [35], (2019)	Greece	Heart failure	System validity testing	ML (RF, logistic model trees, J48, rotation forest, SVM, radial basis function network, Bayesian network, naïve Bayesian, and simple CART^o^)	ML algorithms–based system (HEARTEN Knowledge Management System) classifies patients’ NYHAp class, predicts adverse events, and estimates treatment adherence and the adherence risk regarding medication and overall.	Prediction accuracy of the knowledge management system ranged from 0.78 to 0.95 depending on the algorithms and modules.
Persell et al [77], (2020)	United States	Hypertension	Field testing (2-group RCT)	Conversational AI	Conversational AI–based on proprietary algorithms provides support and tailored coaching to improve self-management and healthy behaviors.	After 6 mo, self-confidence in controlling blood pressure in the intervention group (n=144) was greater than in the control group (n=153). There was no difference in the effect of reducing blood pressure between the groups.
Apergi et al [76], (2021)	United States	Heart failure	Usability testing	Conversational AI	AI collects data regarding treatment compliance and symptoms and generates 3 types of flags based on the risk. Patients are encouraged to maintain their current medications, diet, and activities or advised to follow specific suggestions depending on flags.	Older age, a lower number of medications, and being non-Black are associated with higher use of this technology.
Gomez-Garcia et al [59], (2021)	Colombia	Cardiovascular disease	Laboratory function testing	DL (deep neural network) and ML (logistic regression and RF)	ML algorithms were used to predict the presence or not of cardiovascular risk by processing medical records. The DL algorithm using deep neural networks detects heart arrhythmias by mapping a sequence of electrocardiogram waves to a sequence of rhythm classes.	F-measures were high in the 2 models, 0.83 in the logistic regression classifier and 0.81 in the RF classifier. The F-measure of deep neural network was 0.83.
Luštrek et al [18], (2021)	Slovenia	Heart failure	Field testing (proof-of-concept RCT)	ML (decision tree, k-nearest neighbor, naïve Bayesian, multilayer perceptron, RF, and SVM)	ML algorithms–based system (HeartMan) estimates continuous blood pressure, monitors physical activity using the acceleration data, or recognizes motivated, anxious, and depressed feelings from voice and heart rate. DSS provides exercise plans based on data for patients’ recommendations tailored to patients’ psychological profiles.	The mean absolute error of blood pressure estimation was 9.0/7.0 mm Hg systolic and diastolic blood pressure. F-measure for physical activity recognition was 0.71. the prediction accuracy of the psychological profile was 0.89. Participants’ (n=56) self-care behavior was significantly improved, and rates of depression, anxiety, and perceived sexual problems were reduced.
Chaix et al [53], (2019)	France	Breast cancer	Laboratory function testing	Conversational AI	A chatbot (ViK) generates medication reminders and provides personalized responses by interacting with patients. Patients can build up emotional attachments with the chatbot, contributing to their improved quality of life.	The average medication compliance of patients using the reminder function significantly improved by more than 20%.
Kataoka et al [79], (2021)	Japan	Lung cancer	Usability testing	Conversational AI	A chatbot provides appropriate responses to patients about unfamiliar symptoms that they experienced.	Among 60 questions provided to participants (n=12), 8 (13%) did not match the appropriate topics. The average score of satisfaction was 2.7 out of 5.
Leung et al [81], (2022)	Canada	Cancer	Usability testing	NLP	AI-based on-facilitator system (CancerChatCanada) using NLP technology identifies keywords related to patients’ psychosocial concerns and recommends appropriate resources addressing each concern.	Prediction accuracy was 0.797, recall was 0.891, and the F1-score was 0.880.
Schmitz et al [84], (2023)	United States	Breast cancer	Field testing (2-group RCT)	Conversational AI	A virtual assistant using the Amazon Echo Show with Alexa provides tablet-based supportive care software (Nurse AMIEq). Nurse AMIE monitors lifestyle behaviors, symptoms, and emotional distress and provides timely recommendations.	Participants (n=42) reported high levels of acceptability, feasibility, and satisfaction. There were no significant effects on psychosocial distress, pain, sleep disturbance, fatigue, physical function, or quality of life.
Tawfik et al [85], (2023)	Egypt	Breast cancer	Field testing (3-arm RCT)	Conversational AI	A knowledge-based chatbot (ChemoFreeBot) interacts with patients regarding chemotherapy-related self-management and side effects through the WhatsApp app.	Participants in the intervention group (n=50) had significantly fewer, less severe, and less distressing symptoms compared to nurse-led education (n=50) and the control group (n=50).
Buchan et al [86], (2024)	United States	Cancer	Field testing (1-group pretest-posttest design)	Conversational AI	An AI-based virtual platform (Ina) provides ongoing personalized nutritional and symptom guidance via SMS text based on patient-reported data. A team of live oncology-credentialed dietitians confirms or modifies the guidance if needed.	94% were satisfied with the platform, and 98% reported that the guidance was helpful. 84% and 47% used the advice to guide diet and recommended recipes, respectively. 82% and 88% reported improved quality of life and symptom management, respectively.
Kim and Park [87], (2024)	South Korea	Gastric cancer	Usability testing	Conversational AI	A knowledge-based question-answering chatbot (GastricFAQ) provides real-time answers for patients’ self-management after curative gastrectomy.	The overall mean usability score was 4.28 out of 5 (n=56). The chatbot’s accuracy and F score were 85.2% and 92%, respectively.
Lau-Min et al [88], (2024)	United States	Gastrointestinal cancer	Usability testing	Conversational AI	A mobile phone text messaging-based chatbot (PENNY-GI) provides tailored medication reminders and promotes medication adherence and toxicity management.	Less than 10% of medication or symptom-related messages were identified as incorrect recommendations for participants (n=40). Participants reported that medication reminders are useful but found symptom management tools too simple to be helpful.
Lo et al [47], (2018)	China	Chronic pain	Field testing (1-group pretest-posttest design)	DL (artificial neural network)	The multilayered perceptron artificial neural network was used to learn from historical examples, analyze nonlinear data, and hand imprecise information. An AI algorithms–based mobile app (Well Health) provides the most appropriate therapeutic exercise program by processing data input from patients’ subjective symptom assessment.	Participants (n=161) reported reduced median pain scores from 6 (IQR 5-8) to 4 (IQR 3-6) after using this app.
Nijeweme-d’Hollosy et al [36], (2018)	Netherlands	Chronic pain	Laboratory function testing	ML (decision tree, RF, and boosted tree)	ML algorithms provide referral advice based on patients’ data to help patients manage their low back pain timely.	Prediction accuracy of 3 models ranged from 0.53 to 0.72 depending on the algorithms and dataset. A model using boosted tree was the best for predicting referral advice (κ 0.2-0.4).
Rabbi et al [43], (2018)	United States	Chronic pain	Laboratory function testing	ML (reinforcement learning)	The algorithm using reinforcement learning was used to address the task of being continuously adaptive. The ML algorithm–based system (MyBehaviorCBP) analyzes self-reported physical activity logs and is used to generate personalized physical activity recommendations based on the past behaviors of patients.	Participants (n=10) reported increased walking time for a further 4.9 min/d after receiving recommendations from a 5-wk pilot study. There was no difference in the effect of reducing chronic back pain according to recommendations.
Sandal et al [63], (2021)	Denmark, Norway	Chronic pain	Field testing (2-group RCT)	ML (case-based reasoning)	Case-based reasoning system (selfBACK), a branch of knowledge-driven AI, provides weekly personalized self-management recommendations and motivates patients to perform desired behaviors.	The adjusted mean difference in RMDQr score between groups was 0.79 at 3 mo, favoring the intervention group after 3 mo.
Meheli et al [78], (2022)	United States	Chronic pain	Laboratory function testing	Conversational AI	The Wysa app, using an anonymous conversational AI, uses a free text conversational interface to listen and respond to patients’ distress by providing evidence-based recommendations.	Patients who used the Wysa app were reported to have improvements in means for depression and anxiety symptom scores with a medium effect size (Cohen d=0.60-0.61).
Piette et al [19], (2022)	United States	Chronic pain	Field testing (2-group RCT)	ML (reinforcement learning)	An intelligent agent used reinforcement learning to learn to progressively refine decisions based on probabilistic trials of new choices with feedback about the response. The intelligent agent adjusts the modality of therapist interactions and provides recommendations based on response.	A greater portion of participants in the intervention group (n=168) reported improvement in 6 mo in RMDQ (37% vs 19%) and pain intensity (29% vs 17%) than the control group (n=110).
Barreveld et al [73], (2023)	United States	Chronic pain	Field testing (prospective, multicenter, single-arm clinical trial)	DL (artificial neural network)	The cloud-based AI app (PainDrainerTM) provides digital information material daily via a tablet, smartphone, or computer. The AI engine analyzes patient-reported data regarding pain, sleep, work, physical activity, leisure time, and housework; predicts pain levels; alleviates pain; and increases psychological flexibility.	Participants reported significantly reduced pain interference scores at 6 wk (n=41) and sustained at 12 wk (n=34). 57.5% and 72.5% of participants demonstrated statistically significant MIDs for pain and physical function, respectively. 100% and 81.3% of participants demonstrated an improvement in depression and anxiety scores, respectively.
Marcuzzi et al [65], (2023)	Norway	Chronic pain	Field testing (multiarm parallel-group RCT)	ML (case-based reasoning)	A knowledge-based AI decision support app (SELFBACK) provides weekly tailored self-management recommendations for physical activity and exercise and motivates the desired behavior.	The AI-based app adjunct to usual care did not significantly improve musculoskeletal health compared to control groups at 3 mo (n=294).
Hezarjaribi et al [42], (2016)	United States	Patients taking medications	Laboratory function testing	ML (decision tree)	ML algorithm monitors medication adherence by tracking wrist motions recognized as a logical sequence of motions.	Prediction accuracy in adherence detection was 0.78 with only 1 sensor worn on either of the wrists.
Roy et al [57], (2017)	Canada	Patients taking medications	System validity testing	Others	ML using activity recognition, developed as possibilistic network classifiers, was used to monitor patients’ daily activities, infer whether they took medications, and provide reminders. The agent encouraged patients to maintain appropriate behaviors or change inappropriate behaviors by evaluating adherence and sending messages.	Prediction accuracy ranged from 0.73 to 0.84 for taking medication, pills, and getting water.
Krumm et al [37], (2018)	Germany	Anticoagulation therapy	System validity testing	ML and DL	2 ML-based approaches were selected to test: model predictive control and neural networks using a simple feed-forward network. ML approaches predict and recommend the next dosage of anticoagulation medication by tracking data, including the INRt value.	The mean squared error between recommended and real dosage in the models ranged from 0.0297 to 0.4711.
Blusi and Nieves [56], (2019)	Sweden	Patients taking medications	Usability testing	Others	Intelligent AI implemented in an augmented reality headset manages information related to medication plans, restrictions, and patient preferences and sensor input data to help patients select the right medication and dispense pills in a pillbox following the prescription.	Participants reported the technology was acceptable and feasible. Older age was associated with less comfort and more hesitancy in using the technology.
Zhao et al [70], (2021)	United States	Patients taking medications	Laboratory function testing	DL (neural network)	AI algorithm using neural networks detects medication administration and whether the patient has followed the required steps of handing the medication device and generates an alert if needed.	Insulin pen administration events were detected with 87.58% sensitivity and 96.06% specificity. Inhaler administration events were detected with a 91.08% sensitivity and 99.22% specificity.
Munoz-Organero et al [38], (2016)	United Kingdom	Stroke	Laboratory function testing	ML (J48, EM^u^ clustering)	The J48 classification algorithm and EM clustering were used to classify walking patterns. ML algorithms analyze and classify patients’ walking strategies, and the data is translated into feedback to help patients with stroke effectively self-manage rehabilitation.	In a 10-m walk test (repeated 6 times), there were significant differences in interstride variation computed in this study between stroke survivors (n=14) and healthy control groups (n=10).
Labovitz et al [51], (2017)	United States	Stroke with anticoagulation therapy	Field testing (2-group RCT)	DL (neural network)	An AI platform using neural network (AiCure) identifies the patient, medication, and confirmed ingestion and provides reminders and dosing instructions.	The average cumulative adherence was 90.5%. After a 12-wk study, adherence based on plasma drug concentration levels was 100% in the intervention group (n=15) and 50% in the control group (n=12).
Lin et al [45], (2015)	Taiwan	Frozen shoulder	Usability testing	DL (Propagation neural network)	The DL algorithm using a back propagation neural network calculates motion data measured by wearable sensors and is applied in the motion recognition procedures of sensors for the rehabilitation of patients with frozen shoulders.	Prediction accuracy ranged from 0.60 to 0.95 according to types of exercise.
Belliveau et al [39], (2016)	United States	Spinal cord injury	System validity testing	DL (artificial neural networks) and ML (logistic regression)	2 algorithms were selected to test: logistic regression and artificial neural networks. ML algorithms predict longer-term functional outcomes and independence for self-care activities at the time of hospital discharge in patients with spinal cord injuries.	Prediction accuracy was similar in the 2 models, as it was >0.85 for ambulation status and ranged from 0.76 to 0.86 for nonambulation outcomes.
Hezarjaribi et al [54], (2019)	United States	Chronic conditions	Usability testing	NLP	NLP-based system (EZNutriPal) extracts dietary information and monitors nutrition intake for patients with chronic diseases from speech and free text.	Prediction accuracy in calorie intake estimation was 89.7%.
Wang et al [80], (2020)	China	Multiple chronic diseases	System validity testing	NLP	NLP is used in health recommender systems to provide tailored educational materials for patients with chronic diseases.	The system can achieve a macro precision of up to 0.970 and overall mean average precision scores of up to 0.628.
Jactel et al [64], (2023)	United States	Inflammatory bowel diseases, irritable bowel syndrome	Laboratory function testing	ML	The ML algorithm based on supervised learning (a combination of gradient descent, regularization, and recursive elements) was selected to test. The ML algorithm predicts trigger foods associated with adverse symptoms and is used to provide personalized elimination diets for patients.	After 9 wk, 67.6% of total participants (n=39) reported total symptomatic resolution after the study. There was significant improvement in symptoms and quality of life.
Morato et al [89], (2023)	Brazil	Patients with ostomy	Usability testing	Conversational AI	The AI chatbot (ESTOMABOT) communicates with patients about ostomy management via web chat interfaces.	The overall usability score of the chatbot was 81.5, showing excellent usability.
Cheng et al [90], (2023)	Taiwan	Chronic kidney disease	Field testing (1-group pretest-posttest design)	Conversational AI	The AI chatbot (PD AI Chatbot), combined with social media (LINE Application), provides a patient interface that includes content regarding peritoneal dialysis management, clinical reminders, diet, and resources.	The average satisfaction scores were 4.5 out of 5 (n=297). Infection rates of exit site and tunnel infection were reduced (P=.049 and .02). The peritonitis rate decreased from 0.98 to 0.8 per 100 patient months.
Liu et al [66], (2023)	China	Chronic kidney disease	Laboratory function testing	ML (optical character recognition)	An AI-based mobile app (KidneyOnline) provides interpretation of disease conditions, lifestyle guidance, regular check-ups, early warnings, real-time answers, and clinical reminders based on patient-reported data. Patients take photos of their medical records, test results, and clinical prescriptions and upload them onto the mobile app.	The KidneyOnline app reduced the risk of composite kidney outcome and the mean arterial pressure.
Au et al [83], (2023)	Australia	Chronic liver disease	Usability testing	Conversational AI	The AI chatbot (Lucy LiverBot) provides targeted health information regarding disease, medication, and nutrition and monitors health behaviors via tablet. The chatbot acts as a social companion and improves patient engagement and self-management.	Lucy LiverBot was perceived as a reliable source of information. Participants identified the chatbot as a potential educational tool and device that could act as a social companion to improve emotional well-being.

^a^AI: artificial intelligence.

^b^ML: machine learning.

^c^SVM: support vector machine.

^d^RF: random forest.

^e^DL: deep learning.

^f^RCT: randomized controlled trial.

^g^HbA_1c_: glycated hemoglobin.

^h^Others represent nonspecified AI technologies.

^i^NLP: natural language processing.

^j^AdaBoost: adaptive boosting.

^k^DSS: decision support system.

^l^ABBA: adaptive basal-bolus algorithm.

^m^DRL: deep reinforcement learning.

^n^COPD: chronic obstructive pulmonary disease.

^o^CART: Classification and Regression Trees.

^p^NYHA: New York Heart Association.

^q^AMIE: Addressing Metastatic Individuals Everyday.

^r^RMDQ: Roland Morris Disability Questionnaire.

^s^MID: minimal important difference.

^t^INR: international normalized ratio.

^u^EM: expectation-maximization.

#### Medical Self-Management

Most studies (45/66, 68%) used AI technologies to support the medical self-management of patients with chronic conditions. Four categories of self-management supporting functions include (1) personalized recommendations for medication or treatment-related decision-making, (2) promoting medication adherence and safety, (3) predicting physiological indicators or clinical outcomes, and (4) specific disease management, such as cancer.

First, AI technologies were used to provide patients with personalized recommendations for medication or treatment-related decision-making (13/45, 29%) [19,36,37,44,49,52,55,66,67,83,89,90,92]. AI-based systems recommended daily insulin basal rates, prandial insulin doses, or insulin bolus doses for patients with diabetes [44,49,67] and the next medication dosage for patients receiving anticoagulation therapy [37]. These AI systems used ML and DL technologies to optimize real-time medication adjustments. For example, algorithms based on neural networks or RL were used to tailor insulin dosages in continuous glucose monitoring. In addition, AI-based mobile systems were used to provide personalized coaching and feedback based on glucose levels through a continuous glucose monitoring system or on patient-reported health data (eg, blood glucose, ketonuria, diet, blood pressure, and physical activity) among patients with diabetes [55,92]. ML algorithms, including RL, DT, and RF, were tested to provide referral advice or adjust the modality of therapist interaction among patients with chronic pain [19,36]. A mobile app (KidneyOnline [66]) used optical character recognition to extract data from patient-uploaded photos of medical records and provided tailored clinical reminders among patients with chronic kidney disease. Finally, 4 (31%) studies used AI chatbots integrated with social media platforms or web chat interfaces to provide personalized recommendations for managing specific disease conditions, including exacerbations of COPD [52], chronic liver disease [83], peritoneal dialysis [90], and ostomy care [89]. Most studies (9/13, 69%) in this category were at the stages of evaluations of system validity or human-computer interaction. Only 31% (4/13) of the studies conducted field testing [19,55,90,92].

Second, AI technologies were used to promote medication adherence and safety (8/45, 18%) [42,46,51,56,57,68,70,75]. For example, DL algorithms based on neural networks detected insulin adherence using continuous glucose monitoring and injection data [68] and inhaler administration by audio signal [46]. AI-based systems with ML algorithms adopted DT and activity recognition to monitor and detect medication adherence by tracking patients’ wrist motions [42] or patients’ daily activities [57]. Furthermore, 2 (25%) studies used neural networks to improve medication adherence and safety by detecting medication administration patterns, identifying whether the patient followed the appropriate medication device handling steps, and providing reminders and instructions about dosage [51,70]. An intelligent virtual assistant–based system (VASelfCare [75]) supported medication adherence by interacting with patients with diabetes. Finally, an intelligent agent implemented in an augmented reality headset helped patients select the right medication and dispense pills as prescribed among patients taking complex medication regimens [56]. Most studies (7/8, 88%) in this category were at the stage of evaluations of system validity or human-computer interaction. Only 1 study conducted field testing [51].

Third, AI technologies were used to predict patients’ physiological indicators or clinical outcomes (19/45, 42%) [18,30-35,40,48-50,58-62,72,73,76]. Most studies used ML and DL technologies, such as multiple ML algorithms, including linear regression, logistic regression, RF, SVM, and adaptive boosting, to predict either blood glucose levels or hypoglycemia events based on patients-reported health data (eg, diet, blood glucose, or medication) or continuous glucose monitoring data. For example, DL algorithms based on neural networks were used to predict blood glucose levels among patients with diabetes by analyzing patients’ intake of carbohydrates, physical activity, or weight [48-50,72]. A mobile-based AI system (forDiabetes [60]) used ML and DL technologies to predict next-day hypoglycemia events in daily life based on the data input from a mobile app and portable devices. Studies among patients with respiratory diseases also tested multiple ML algorithms, including SVM, RF, and adaptive boosting, to predict the risk of asthma exacerbation and generate early warnings of aggravation based on patient health data, such as respiratory symptoms, sleep, physical activity, medication, and measured peak expiratory flow [31-34,58]. An interactive cloud-based digital app (myCOPD [62]) predicts exacerbations of COPD before 1 to 8 days based on patient-reported data. In addition, ML and DL algorithms were tested to predict adverse events and continuous blood pressure, classify the extent of heart failure, identify heart arrhythmia among patients with cardiovascular disease [18,35,59], and predict pain levels in patients with chronic pain [73]. Only 1 study used conversational AI to predict heart failure risk based on collected data from patients regarding treatment adherence and symptoms [76]. Most studies (14/19, 74%) in this category were at the stage of evaluations of system validity. Some (5/19, 26%) studies conducted evaluations of human-computer interaction [49,59,76] or field testing [18,73].

Finally, AI technologies were used for specific disease management, such as cancer management (6/45, 13%) [53,79,84,85,87,88]. All studies used conversational AI, such as a chatbot or a virtual assistant. Chatbots were reported to support the management of oral anticancer agents and cancer treatment–related symptoms by providing medication reminders, promoting medication adherence, and managing toxicity [53,79,88]. Knowledge-based chatbots were developed and tested with patients to manage chemotherapy-related side effects management via the WhatsApp (Meta Platforms) app and to provide real-time question-answering support for patients after curative gastrectomy [85,87]. Finally, a virtual assistant implemented in a tablet supported symptom management and provided timely recommendations for patients with breast cancer [84]. Most studies (4/6, 67%) in this category were at the stage of evaluations of human-computer interaction. Only 2 (33%) studies conducted field testing [84,85].

#### Behavioral Self-Management

Over one-third of the studies (27/66, 41%) used AI technologies to assist in the behavioral self-management of patients with chronic conditions. Two categories of behavioral self-management support include (1) personalized recommendations and feedback on patients’ lifestyles and healthy behaviors and (2) predicting and monitoring health behavior outcomes.

Most of the studies (21/27, 78%) fell into the first category, offering personalized recommendations and feedback on patients’ lifestyles and healthy behaviors [17,18,41,43,47,54,63-66,74-77,80,82,84,86,90-92]. Various AI technologies, such as conversational AI, NLP, ML, and DL, were used to provide personalized support related to diet, physical activity, and other lifestyles for patients with chronic conditions. For example, conversational AI–based systems, such as chatbots and intelligent virtual assistants, offered tailored support based on interactions with patients and the analysis of their previous data, particularly for those with diabetes [17,74,75,82]. Conversational AI was also used to make recommendations regarding diet or physical activity based on patient-reported free text or speech among patients with cardiovascular diseases [76,77] and multiple chronic conditions [54,80].

AI-based virtual assistant platforms supported lifestyle behaviors and nutrition monitoring via patient-reported data submitted through tablets or SMS text messaging among patients with cancer [84,86]. An AI chatbot (PD AI Chatbot [90]) used in conjunction with a social media application provided diet information tailored to patients with chronic kidney disease undergoing peritoneal dialysis. In addition, AI systems delivered recommendations to help patients track their sleep, physical activity, or other health behaviors via mobile apps [66,92]. These systems also offered nutritional support by translating ML algorithms outputs concerning meal patterns and blood glucose levels [91].

An ML algorithm using the SVM classifier implemented in a smartphone app was tested to classify food types and volumes, thereby calculating carbohydrates to aid diet management for patients with diabetes [41]. A supervised learning-based ML algorithm was explored to tailor diets for patients with inflammatory bowel diseases or irritable bowel syndrome by analyzing the association between trigger foods and adverse symptoms [64]. The AI-based system (HeartMan [18]) evaluated multiple ML algorithms, including DT, RF, and SVM, to monitor physical activity using acceleration data, providing personalized exercise plans among patients with heart failure. Moreover, ML algorithms, such as RL and case-based reasoning, were tested to deliver customized physical activity recommendations based on patient-reported data and activity logs for those with chronic diseases [43,63,65]. Finally, a mobile-based AI app (Well Health [47]) used a multilayered perceptron artificial neural network to analyze and process data from patients’ subjective symptom assessment, offering appropriate therapeutic exercise programs for patients with chronic pain. Studies (17/21, 81%) in the first category of behavioral self-management support were at the stage of evaluations of human-computer interaction or field testing. Only 4 (19%) studies remained at the stage of system validity testing [41,75,76,80].

In the second category, AI technologies, primarily ML and DL algorithms, were used to predict and monitor patients’ health behavior outcomes (8/27, 30%) [18,35,38,39,45,69,71,84]. For instance, multiple ML algorithms, including RF, SVM, and DT, were tested to predict treatment adherence, adherence-related risks, or physical activity among patients with heart failure [18,35]. A DL algorithm using artificial neural networks was used to process data on symptom self-management ability, classifying it into 3 levels among patients with COPD [69]. ML algorithms using J48 and expectation-maximizataion clustering, along with a neural network trained using backpropagation, were used to monitor rehabilitation by analyzing walking strategies or calculating motion data from wearable sensors among patients with stroke [38] and frozen shoulders [45]. ML algorithms based on logistic regression and artificial neural networks were tested to predict ambulation status and independence at hospital discharge among patients with spinal cord injuries [39]. An AI-based mobile platform (Auto-Check Care [71]) used a convolutional neural network to integrate diet and nutritional data from photographs taken by patients with diabetes. Only one study used a conversational AI–based virtual assistant to monitor physical activity among patients with breast cancer [84]. Studies (5/8, 63%) in this category focused on evaluating system validity or human-computer interaction, while only 3 (37%) of studies conducted field testing [18,71,84].

#### Emotional Self-Management

A few studies (14/66, 21%) used AI technologies to support the emotional self-management of patients with chronic conditions. Two categories of emotional self-management support include (1) providing personalized emotional support and (2) motivating patients to perform self-management.

AI technologies were used to provide personalized support for emotional psychosocial concerns (9/14, 64%) [18,52,53,73,75,78,81,83,84]. Several studies used conversational AI technologies, such as virtual assistants, chatbots, and NLP, to encourage emotional expression, build emotional attachments, identify psychosocial concerns, and help deal with psychosocial concerns among patients with diabetes [75], COPD [52], chronic liver disease [83], chronic pain [78], and cancers [53,81,84]. In addition, 2 (14%) studies used ML and DL technologies to recognize emotions and manage psychological well-being. An AI-based decision support system (HeartMan [18]) tested multiple ML algorithms to recognize motivated, anxious, and depressed feelings from the voice and heart rate of patients with heart failure. A cloud-based AI application (PainDrainerTM [73]) used artificial neural networks to analyze patient-reported data regarding pain, sleep, work, physical activity, leisure time, and housework to manage pain and increase psychological flexibility among patients with chronic pain. Most studies (8/9, 89%) in this category were at the stage of evaluations of human-computer interaction or field testing. Only 1 study remained at the stage of system validity testing [75].

In addition, AI technologies, such as conversational agents and ML technologies, were used to motivate patients to perform self-management (6/14, 42%) [17,52,57,63,65,74]. Studies used conversational AI to encourage patients to perform self-management by communicating with patients and providing motivational messages to reduce difficulty in conducting specific tasks among patients with diabetes [17,74]. An AI chatbot (Avachat [52]) was tested to provide motivational support for patients with COPD to engage in general self-management during periods of low moods. A knowledge-based AI decision support app (selfBACK [63,65]) used case-based reasoning to provide tailored self-management recommendations for patients with chronic pain and motivate and reward them for following the recommendations. Finally, an ambient intelligent system used a multiagent activity recognition approach to monitor and motivate patients’ self-management activities, such as medication adherence [57]. Most studies (4/6, 67%) in the category of emotional self-management were at the stage of evaluations of system validity or human-computer interaction. Only 2 (33%) studies conducted field testing [17,63,65].

## Discussion

### Principal Findings

To the best of our knowledge, this study is the first to provide a comprehensive overview of AI applications for self-management of chronic conditions, categorizing them according to their developmental stage based on 3 essential self-management tasks: medical, behavioral, and emotional self-management. Our review indicates that most studies have concentrated on enhancing medical or behavioral self-management tasks, and fewer focus on emotional self-management supported by AI. In addition, the current stage of AI applications for chronic condition self-management largely remains in the algorithm development and early feasibility testing phases, except for providing lifestyle recommendations and personalized emotional support. Among chronic conditions, diabetes was the most frequently studied, with the primary focus of most studies on evaluating the prediction accuracy and validity of the algorithms. Meanwhile, AI-based interventions have been relatively more developed for conditions targeting chronic pain management using ML and DL techniques, as well as for conditions where conversational AI supports self-management among patients with cancer. This study has expanded on previous research by identifying how AI supports self-management, focusing on specific tasks and categorizing the application of AI support for chronic condition self-management into technology development stages.

Advancements in AI technologies provide a significant opportunity to empower patients to effectively perform essential self-management tasks and enhance the quality of life in home settings by fostering patient engagement in managing chronic conditions [48,93-95]. Our review confirmed the capability of AI, enabling patients with various chronic conditions to make informed day-to-day decisions about managing their diseases based on AI-generated solutions and shared information [96]. In addition, findings from the field testing of AI technologies revealed the potential effectiveness of AI applications for self-management in the real world. For instance, most RCTs reported significant effectiveness of AI-based interventions on improved health outcomes, including blood glucose levels, pain, symptom distress, treatment adherence, and quality of life [17-19,51,63,71,85]. This suggests that AI applications in managing chronic conditions have not only augmented patients’ self-management capabilities but also ensured a more proactive and comprehensive care model.

There are several potential interpretations of the early development stage and testing of AI technology for chronic condition self-management. Our review highlighted that many studies using AI technologies to predict physiological indicators or clinical outcomes, such as blood glucose levels or the risk of adverse events, primarily focused on algorithm development. The types and performance of these algorithms vary across the studies. Most validation studies predicting blood glucose or hypoglycemic events among patients with diabetes are frequently conducted, with prediction accuracy of ML and DL algorithms reported ranging from 63% to over 90% [30,40,72]. ML algorithms, including SVM, RF, and adaptive boosting, are often used to predict the risk of asthma exacerbation; however, their prediction accuracy varies from 79% to 86% across different studies [31,32,34]. Given the complexity of individual health indicators, AI technologies may struggle with limited data input [48]. For example, distinguishing between medication-related side effects and symptoms of underlying diseases and comorbidities could be challenging for both AI and humans [2]. Therefore, integrating AI-collected health data with additional predictive factors (such as genetic traits, clinical variations, or sociodemographic characteristics) could effectively enhance the accuracy of prediction by leveraging extensive data streams [31,48]. Moreover, individual differences and lifestyle variations may further complicate predictions [69], suggesting the need for a comprehensive approach to multiple self-management tasks when applying AI technologies for chronic condition self-management.

Technological or implementation challenges may also contribute to the early developmental stages of AI applications in chronic condition self-management. Key technological barriers include cost, accessibility, and interoperability between devices. For instance, patients might have concerns about whether AI-based services are covered by insurance or involve out-of-pocket expenses [95]. Interoperability involves customizing AI technologies’ delivery modalities to meet user requirements and support various types of technology. For example, the benefits of conversational AI could be significantly enhanced by personalization and the capability to interact with a range of digital and domestic devices, such as calendars, smart home technologies, or medical devices [52]. In addition, dataset-related issues, such as imbalanced or limited datasets, pose significant challenges to the implementation and generalization of AI systems, potentially introducing bias in decision-making [97]. Adopting balanced evaluation metrics and data-driven algorithmic models may help mitigate this potential bias [97].

An important consideration in AI applications for chronic disease management is ensuring data security and privacy, which may be achieved through a robust implementation framework [98]. Traditional ML models, which rely on computational power and the volume of training data from centralized servers, often face challenges related to the security and privacy of patient data [99]. These concerns can limit usability and result in nonparticipation in studies due to patient-level barriers [85,88]. Federated learning offers a transformative solution by enabling organizations to collaboratively analyze massive datasets without compromising sensitive patient information [99]. In addition, federated learning can enhance security when integrated with technologies such as blockchain, which provides an immutable ledger for storing and preserving information [99,100]. Furthermore, the nonadoption, abandonment, scale-up, spread, and sustainability, developed by Greenhalgh et al [101], provides principles for implementing AI techniques in health management. Future studies should focus on leveraging these technologies and frameworks to develop and implement AI algorithms that ensure robust data privacy while enhancing chronic disease self-management.

The chronic nature of many health conditions often leads patients to experience emotional distress, such as depression, anxiety, or feelings of isolation [95,102]. Despite the significance of emotional self-management for individuals with chronic conditions [8], our findings indicate a lack of focus on emotional aspects in current AI applications. Several factors could contribute to this gap. First, the variability in mental health status means that the criteria for identifying emotional self-management are not specific enough to produce AI algorithms with high sensitivity and specificity [103]. Second, developing effective AI systems require extensive training and validation using large datasets [94]. The difficulty in accessing comprehensive and high-quality mental health datasets may hinder studies aimed at AI-based emotional support [103]. One viable strategy to address dataset limitations is to leverage transfer learning, which uses pretrained algorithms to develop AI systems that support emotional self-management [103,104]. In addition, some patients may prefer direct interaction with health care providers for managing emotional distress or may lack the motivation to engage with AI solutions. Therefore, a blended model that integrates face-to-face support with AI-based interventions might be more acceptable and effective than relying solely on AI [52,105]. It is crucial for AI-based systems to emulate key aspects of human interaction and provide tailored support aligned with person-to-person care based on comprehensive needs assessments [52]. This approach ensures that AI systems are both technically proficient and adaptable to patients’ diverse emotional needs, thereby enhancing their ability to manage emotions effectively.

The evaluation of AI applications and their impact on individuals with chronic conditions reveals a notable lack of uniformity. Usability tests have uncovered a significant gap between the development of AI systems and the challenges associated with transferring algorithms into practical applications. While results from early-stage feasibility tests show promise, research is needed to thoroughly understand user experiences and engagement within everyday living environments. In addition, given that not all individuals are willing to integrate AI technologies into their health care, it is crucial to conduct comprehensive assessments of patients’ needs and attitudes toward AI for successful implementation [106,107]. Several studies have raised concerns about the potential loss of control when AI monitors patients’ lifestyles [2,18], underscoring the importance of designing AI-based interventions that prioritize patient empowerment and autonomy rather than mere supervision. By creating AI-based solutions that enhance patient empowerment and self-efficacy, patients can make health data–based decisions, thereby increasing the objectivity and accuracy of their knowledge without compromising the subjective and authentic aspects of their experience [96]. Furthermore, although AI systems excel at processing numerous data points and delivering data-driven insights for disease self-management, their effectiveness is highly contingent on patient engagement and the accuracy of the provided data. Therefore, further research using user-centered design principles in the system development phase is necessary to ensure that AI-supported self-management components align with patients’ needs and preferences, addressing potential issues of nonadoption or low adherence [94]. In addition to conducting field tests, process evaluations will help to identify barriers and facilitators to the uptake and engagement of AI-based interventions from the patients’ perspectives [2].

### Limitations

Our review has several limitations. Despite including several databases in the search process, the specific choice of search terms may have resulted in some relevant articles being missed, especially considering the rapid study of AI applications across multiple areas. However, the increase in publications over the last 5 years suggests that our search captured a significant period of research and development in AI for self-management. In addition, the developmental stages and outcomes reported in the studies varied, making it a challenge to compare the effectiveness of AI technologies across different studies. Furthermore, we only included studies published in English. As most studies in our review were conducted in high-income countries, our findings may not be generalizable to diverse settings. More extensive studies with various samples are needed to establish evidence on the application of AI across different geographic and cultural contexts.

### Conclusions

AI applications have the potential to empower patients with chronic conditions to effectively perform self-management tasks and enhance their quality of life in home settings. Although most studies are still in the stages of algorithm development or early feasibility testing, and several challenges related to technology implementation were identified, AI can offer personalized medical recommendations, support data-driven treatment decision-making, encourage the adoption of healthy lifestyles, and manage emotional distress associated with chronic condition self-management. This review provides evidence to guide the development and selection of AI solutions for supporting self-management in patients with chronic conditions. However, there is still a long journey ahead to fully integrate AI applications into self-management practices and achieve optimal outcomes.

## Appendix

Multimedia Appendix 1 PRISMA-ScR checklist.

Multimedia Appendix 2 Search strategy.

## Abbreviations

AI: artificial intelligence
COPD: chronic obstructive pulmonary disease
DL: deep learning
DT: decision tree
MeSH: Medical Subject Headings
ML: machine learning
NLP: natural language processing
PRISMA-ScR: Preferred Reporting Items for Systematic Reviews and Meta-Analyses Extension for Scoping Reviews
RCT: randomized controlled trial
RF: random forest
RL: reinforcement learning
SVM: support vector machine
