# Supplementary material for: AI Applications for Chronic Condition Self-Management: Scoping Review
Source: J Med Internet Res. 2025 Apr 8;27:e59632. doi: 10.2196/59632 (PMC12015343; doi:10.2196/59632)
Supplement: Multimedia Appendix 2 [file jmir_v27i1e59632_app2.doc]

**Multimedia Appendix 1.** Search strategy

**1. PubMed**

("Self-Management"[Text Word] OR "Self Care"[Text Word] OR "symptom management"[Text Word] OR "Self-Management"[MeSH Terms] OR "Self Care"[MeSH Terms]) AND ("Machine Learning"[Text Word] OR "Natural Language Processing"[Text Word] OR "Artificial Intelligence"[Text Word] OR "Natural Language Processing"[MeSH Terms] OR "Machine Learning"[MeSH Terms] OR "Artificial Intelligence"[MeSH Terms])

**2. Web of Science**

TS=((“Machine Learning” OR “Natural Language Processing” OR “Artificial Intelligence”)) AND TS=((“self management” OR “self care” OR “symptom management”))

**3. CINAHL**

(((MH "Self Care") OR (MH "Self-Management")) OR (“self management” OR “self care” OR “symptom management”)) AND (((MH "Natural Language Processing") OR (MH "Machine Learning+") OR (MH "Artificial Intelligence+")) OR (“Machine Learning” OR “Natural Language Processing” OR “Artificial Intelligence”))

**4. PsycINFO**

(((Any Field: ("self management") OR Any Field: ("self care") OR Any Field: ("symptom management"))) AND (((Any Field: ("Natural Language Processing") OR Any Field: ("Artificial Intelligence") OR Any Field: ("Machine Learning")))
